# Comparison of Absorbable and Nonabsorbable Sutures for Intradermal Skin Closure in Dogs

**DOI:** 10.3390/vetsci10020105

**Published:** 2023-02-01

**Authors:** Dimitrios B. Balomenos, Pagona G. Gοuletsοu, Apostolos D. Galatos

**Affiliations:** 1 Clinic of Surgery, Faculty of Veterinary Science, School of Health Sciences, University of Thessaly, Trikalon 224, 43100 Karditsa, Greece; 2 Clinic of Obstetrics and Reproduction, Faculty of Veterinary Science, School of Health Sciences, University of Thessaly, Trikalon 224, 43100 Karditsa, Greece

**Keywords:** canine, intradermal, intradermal with clips, polypropylene, poliglecaprone 25, wound healing

## Abstract

**Simple Summary:**

The choice of suture material for skin closure can affect the final cosmetic outcome, the risk of wound infection and other complications in companion animals. We assessed two commercially available suture materials, namely, Monocryl and Securex, for use in suturing the skin of dogs, using cosmetic, clinical, ultrasonographic and histological evaluations. The results indicated only minimal differences between the two products, although better scores were achieved using Monocryl. Both were found to be sufficient for use in intradermal suturing in dogs. The earlier removal of Securex compared to Monocryl did not have any additional beneficial effect on wound healing and scar appearance in dogs.

**Abstract:**

The study aimed to compare incisional wound healing with intradermal suture patterns performed with (a) absorbable suture with burying of the knots and (b) nonabsorbable suture anchored with clips. Ten dogs were included in the study. Surgically created skin incisions were apposed with continuous intradermal suture pattern with 4/0 poliglecaprone 25 with burying of the knots and continuous intradermal pattern with 4/0 polypropylene with clips. Cosmetic, clinical, ultrasonographic and histological scores were evaluated. The intradermal pattern with clips was easier to perform and required significantly less time to complete than the intradermal suture with burying of the knots. Cosmetic, clinical, ultrasonographic and histological evaluation scores did not differ significantly between the techniques. Irrespective of the technique used, the cosmetic, ultrasonographic, clinical and histological appearances of the incisions improved over time. In conclusion, polypropylene was found to be a safe and effective suture material for use with intradermal suture pattern with clips in dogs and to have an easy and quick application. However, in our sample, its earlier removal from wounds than poliglecaprone 25 was not found to be associated with a supposedly beneficial effect on wound healing and scar appearance. Both suture materials can be useful in intradermal suture techniques in dogs.

## 1. Introduction

Continuous intradermal skin closure has become increasingly popular in both elective and nonelective surgical procedures in small animals [1,2,3,4,5,6]. Advantages of the buried continuous intradermal closure pattern include decreased scar formation because of the promotion of epithelialization due to adequate skin apposition and minimal skin tension, elimination of the need for suture removal, reduction of tissue inflammation and risk of infection by avoiding the formation of percutaneous suture tracts, and reduction of self-induced trauma because no suture material is exposed to the environment [4]. However, the buried continuous intradermal closure is more technically demanding and time-consuming than the traditional percutaneous suture techniques [1,2,3,5]. Moreover, in studies which evaluated the healing of wounds sutured with intradermal suture pattern over time, it was observed that the appearance of wounds could deteriorate subsequent to the first postoperative month, possibly due to skin irritation, caused by the suture material [5,6]. This was observed when absorbable poliglecaprone 25 was used for intradermal suturing and also when absorbable polyglytone 6211 was used, even though the former is absorbed earlier (100 days and 56 days postoperatively, respectively). In order to minimize skin irritation caused by the suture material, monofilament nonabsorbable suture materials have been used for intradermal suture patterns in humans [7,8,9,10]. When a synthetic nonabsorbable suture material is used, the suture material is anchored just before and after the commissure of the wound with two fixation clips or sterile buttons or superficial-suture material loops. No knots are buried in the subcutaneous tissue, thus reducing the amount of foreign material left in the wound site, and the suture is removed 10–14 days postsurgery [4]. There are only a few studies in the veterinary literature comparing absorbable and nonabsorbable monofilament suture materials for continuous intradermal closure in cats [4], rats [11,12] and pigs [13]; however, no such study has been performed in dogs.

The objective of this study was the comparative evaluation of the healing process (by cosmetic, clinical, ultrasonographic and histological evaluations) after employing 4/0 polypropylene without burying of the knot and 4/0 poliglecaprone 25 with burying of the knot in a continuous intradermal suture pattern. Our hypothesis was that the use of 4/0 polypropylene would be superior to 4/0 poliglecaprone 25 for intradermal suturing, given that, as it is removed earlier (12 days versus 100 days postoperatively), it would probably cause less skin irritation at the wound area and achieve better healing outcomes.

## 2. Materials and Methods

### 2.1. Animal Ethics

The study was performed in the research facility area of the Clinic of Surgery, Faculty of Veterinary Science, University of Thessaly. The research protocol of the study was approved by the Animal Ethics Committee of Greece (1174/13.4.2009) as being in accordance with the European Union legislation concerning the welfare of laboratory animals. All legal and ethical requirements concerning the welfare of laboratory animals were met.

### 2.2. Animals

Ten healthy Beagles (5 of each gender), aged 1 to 5 years, were used. During the first month of the study, the animals were housed individually; subsequently, they were separated into three pens, two containing 3 and one containing 4 dogs. For the entire procedure, strict measures regarding pain management and handling were taken, while the experiments were performed according to the laws of the EU.

### 2.3. Inclusion Criteria

All dogs were clinically healthy; no wounds, scars or active skin lesions were evident in the areas where incisions were to be performed. Before the study, the health of each dog was assessed by clinical and laboratory examinations (full haematological and blood and urine biochemical examinations); the examinations were repeated at monthly intervals throughout the study period.

The only pharmaceutical or immunological products administered to the animals 30 days prior to and throughout the study were the following: (a) scheduled vaccines and routine antiparasitic products and (b) antibiotics and opioids, administered during the perioperative period, as detailed below.

### 2.4. Experimental Design

The dogs were premedicated by intramuscular injection of dexmedetomidine (300 μg/m^2^; Dexdomitor, Pfizer Hellas, Greece) and morphine (0.5 mg/kg; Morphina cloridrato, Molteni Pharmaceutici, Florence, Italy). After 30 min, 2.5% thiopental sodium (5–7 mg/kg; Pentothal, Abbott Hellas, Athens, Greece) was administered intravenously for the induction of anaesthesia, and, after intubation of the trachea, anaesthesia was maintained with isoflurane (1.5–2%; Aerrane, Baxter, Norfolk, UK) in oxygen (1 L/min), using a semi-closed anaesthetic circuit. During anaesthesia, Lactated Ringer’s solution (10 mL/kg/hour; Lactated Ringer’s, VIOSER, Trikala, Greece) was administered by continuous intravenous infusion.

The same veterinary surgeon (PGG) performed all of the surgeries. Two 12 cm-long skin and subcutaneous tissue incisions were performed with a Νο. 10 lancet at the lateral aspect of each thigh. After vascular clamping with haemostatic forceps to control bleeding, the subcutaneous tissue was closed primarily with a continuous subdermal suture pattern using 3/0 polyglactin 910 (Vicryl, Ethicon Inc, Bridgewater, NJ, USA). Subsequently, the selected skin-closure technique was performed; the technique to be performed was decided randomly on the spot by selecting a presealed envelope in which the combination to be performed was specified. The procedure was performed initially at the right thigh and subsequently at the left.

The intradermal pattern with burying of the knots was performed with 4/0 poliglecaprone 25 (Monocryl, Ethicon, Inc, Bridgewater, NJ, USA) (intradermal B). The initial knot of the suture material was buried in the subcutaneous tissue at a distance of 4 mm from the commissure of the wound with a square knot (5 throws), and then the suture material was directed towards the start of the incision in the middle of the dermis. The needle was positioned horizontally, through the dermis, with particular care taken to confirm that no material of the suture crossed the epidermis. Approximately 2.5 cm before the end of the wound, the suture was anchored with an Aberdeen knot with four throws, so that a biopsy could be performed for the last 2 cm of the suture line without collapse of the entire suture. At the last passage of the suture from the dermis, the needle was directed backwards, and the suture was fixed with an Aberdeen knot with four throws in the subcutaneous tissue.

The continuous intradermal suture pattern was performed with polypropylene (without burying of the knot, anchored with clips, using 4/0 polypropylene; Securex, B Braun Aesculap, Tuttlingen, Germany) (intradermal C). The suture material was anchored to one end of the skin incision with a fixation clip just before the commissure of the surgical skin incision, and the needle was passed from that point through the wound commissure. Suturing was continued by passing the suture material horizontally at the middle of the dermis. Approximately 2.5 cm before the end of the wound, the suture was anchored with an extra clip, so that a biopsy could be performed at the last 2 cm of the suture line without collapse of the entire suture. On the final bite, the suture material passed through the full thickness of the skin and was secured with a third fixation clip.

The time required to complete each suture was recorded.

### 2.5. Postsurgery Care

After the surgery and until the 10th postoperative day (po.d.), the dogs wore Elizabethan collars, and bandages were applied to their thighs to protect the wound sites. On the 10th po.d., the polypropylene suture material with clips was removed.

The dogs received morphine at a dose rate as above, im, every four hours for three days postoperatively and every six hours for the next two days. Moreover, an amoxicillin/clavulanic acid combination was administered to the animals (Synulox, Haupt Pharma Latina S.r.l., Latina, Italy) at a dose rate of 12.5 mg/kg, sc, twice daily until the 10th po.d.

### 2.6. Cosmetic Evaluation

**Figure 1:**
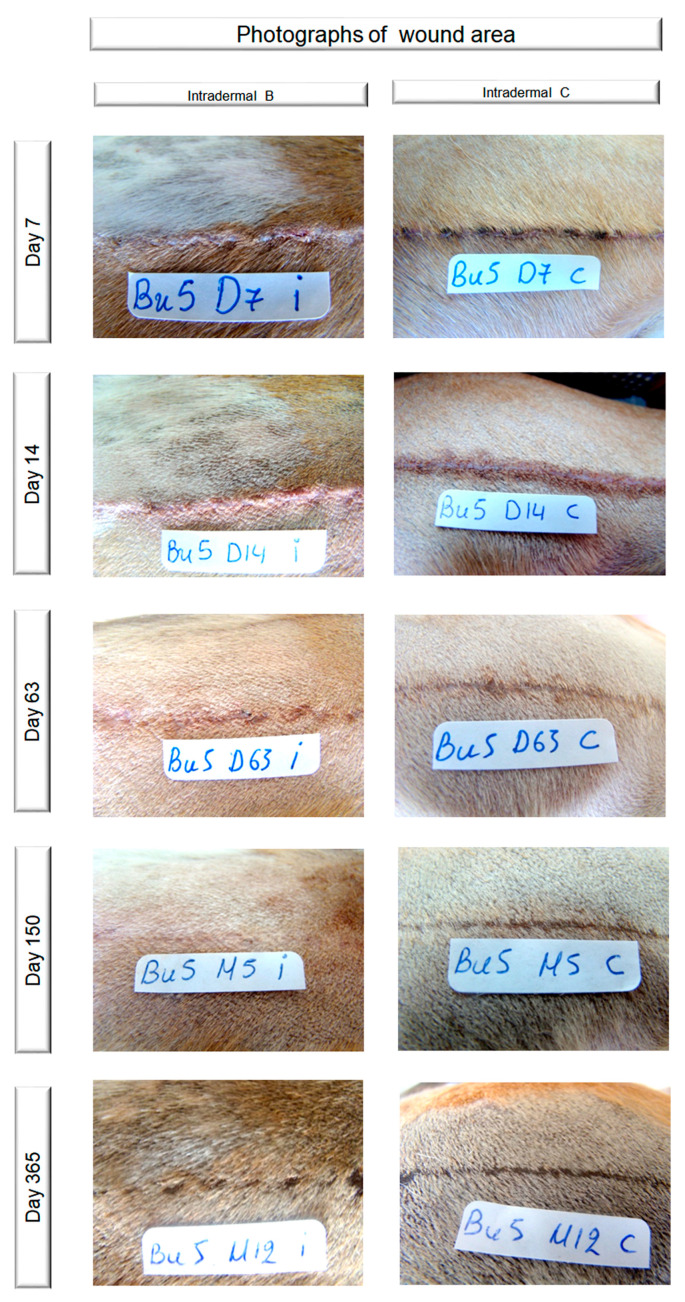
Photographs of the wounds (the sticker is 32 mm long).

Two experienced surgeons (PGG and ADG) blindly evaluated the appearance of the wounds from photographs taken on days 0–10, 12, 14, 16, 18, 21, 24 and 28, once a week until the end of the 3rd postoperative month, and once a month until the end of the experiment, i.e., one year after the incision, using a 1–10 visual analogue scale (1: excellent cosmetic result, 10: bad cosmetic result). The two scores given were averaged to produce a total cosmetic score for the wound. Photographs of the wounds are presented in Figure 1.

### 2.7. Clinical Evaluation

Clinical evaluation was performed immediately after surgical skin closure (Day 0), every day until the 10th po.d., on the 12th, 14th, 16th, 18th, 21st, 24th and 28th po.d., once a week until the end of the 3rd postoperative month, and once a month until the end of the experiment. During clinical evaluation, the following parameters were evaluated, always by the same person (DBB): skin thickening (in mm, using a tuberculin skin testing ruler/skin caliper), erythema (in mm, with an electronic caliper), scar width (in mm, with an electronic caliper), abscessation or inflammation (score 0–3), exudate (score 0–3), comedones (score 0–3), hyperpigmentation (score 0–3), suture loss (score 0–3) and wound dehiscence (score 0–3), according to the scoring system proposed by Gouletsou et al. [5] (Supplementary Materials, Table S1). When skin thickening, erythema or scar width was uneven along the incision, five measurements were taken and the mean value of the five results was used. Normal skin values were assessed before the onset of the experiment and 3 cm caudally from the middle of the incision lines throughout the experiment.

### 2.8. Ultrasonographic Evaluation

**Figure 2:**
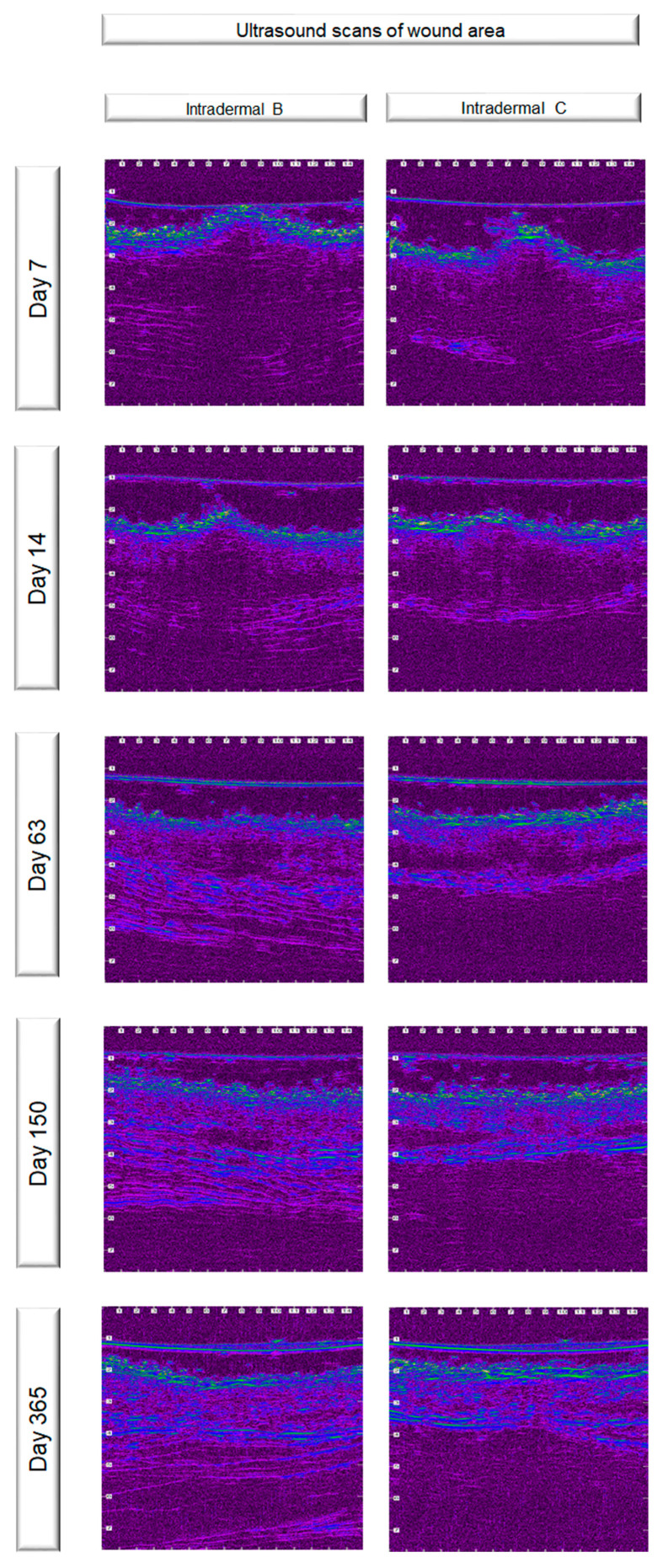
Ultrasound scans of the wound areas (scale in mm).

B-mode real-time ultrasonographic examination of the skin was performed by means of a real-time ultrasound machine. The ultrasound unit (Longport Digital Scanner (LDS1), Longport International Ltd., Silchester, United Kingdom) was fitted with a 50.0 MHz polyvinylidene difluoride transducer incorporated into a probe filled with distilled water and scanned using a digital stepping motor. The ultrasound beam was propagated through an aperture covered with a disposable rubber membrane; a new membrane was used for each wound. The transducer was applied to the wound area using light pressure and coupling gel as a transmission medium. Scans perpendicular to the long axis of the incision and the adjacent intact skin were taken. Four transverse images (a, b, c and d) were taken of each wound, in such a way that the distance between the scans was 2 to 3 cm. Wounds were examined daily until the 10th po.d., on the 12th, 14th, 16th, 18th, 21st, 24th and 28th po.d., once a week until the end of the 3rd month postoperatively, and once a month thereafter until the end of the experiment. Normal skin values were assessed before the onset of the experiment and 3 cm caudally from the middle of the incision lines throughout the experiment. Furthermore, a scan was performed at the area where the skin punch biopsy would be performed, just a few minutes before the biopsy. The digitized scans were stored on the associated hard drive and were visualized using a colour palette (rainbow). Images were compressed laterally to facilitate viewing of the wound areas. Wound area calculations were performed using computer software. The ultrasound scans of the wound areas are shown in Figure 2.

### 2.9. Histological Evaluation

**Figure 3:**
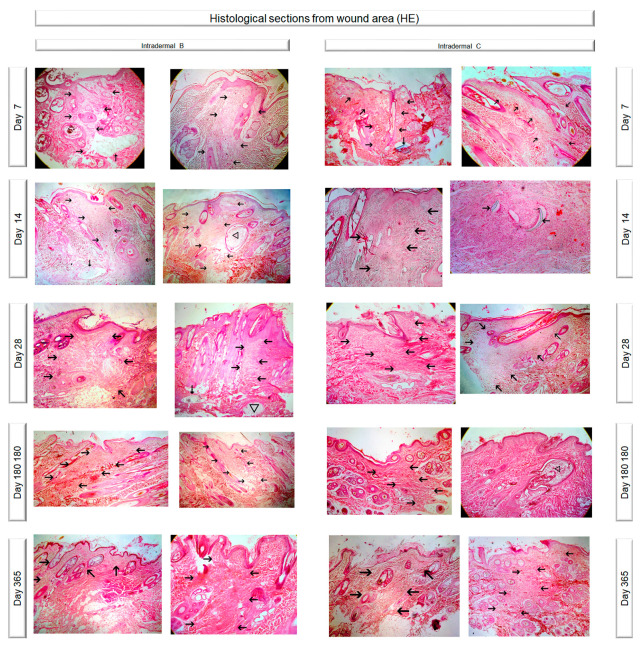
Photographs of the histological sections of the wound areas. (black arrows: wound area at the site of incision; dotted arow: suture material; triangle: traumatic furunculosis)

Histological evaluation of the healing process was performed on postoperative days 7, 14, 28, 180 and 365 [5,6,11,12,14,15]. The sample collection for histological examination was performed at the incision line with a disposable biopsy punch with a diameter of 8 mm [16]. The first sample was taken on the 7th po.d., 1 cm away from the lower commissure of the incision, whilst the others were taken 1.5 cm apart from the previous one, proximally. After the collection of the sample, the wound was closed with a simple interrupted stitch with a 4/0 poliglecaprone 25 suture. The samples were immediately bisected, under magnification, perpendicularly to the incision, fixed in 10% buffered formalin and stained with haematoxylin and eosin. The histological sections were evaluated according to already existing scales [2,5,17,18]. Normal skin was assessed at the onset of the experiment by taking a biopsy sample 3 cm caudally from the middle of the incision lines. During the experiment, the wound area was compared to that of the normal skin at the edge of the same biopsy sample. Photographs of the histological sections of the wound areas are presented in Figure 3.

The following parameters were evaluated: necrosis (score 0–3), oedema (score 0–3), inflammation (score 0–3), epithelial gap (in mm), presence of suture (score 0–3) and tissue reaction around the suture (score 0–3), epithelial thickness (number of times by which the epithelial thickness at the scar was greater than that of the adjacent healthy epidermis), scar width (mm), collagen synthesis (score 0–3), presence of fibroblasts (score 0–3), and angiogenesis (score 0–3). The scoring system is presented in the Supplementary Materials, Table S2.

### 2.10. Statistical Analysis

All the continuous variables were tested against the normal distribution with the Kolmogorov–Smirnov one-sample test of normality. According to the results of the preview tests, the qualitative data are presented as medians (interquartile ranges) and the quantitative data as frequencies and proportions.

For the initial detection of differences between the results for every technique, the Kruskal–Wallis test was used. When the test produced statistically significant results (*p* < 0.05), we performed pairwise comparisons with Mann–Whitney testing, reducing the significance level accordingly with the use of Bonferroni correction.

For intragroup analysis of repeatedly measured variables (separate time intervals), Friedman analysis of variance by ranks was chosen, accompanied by the Wilcoxon test for pairwise comparisons, reducing the significance level, following Bonferroni correction. The level of statistical significance for all comparisons in this study was set at 5%, and all the calculations and tests were performed with IBM SPSS 20 software (IBM, New York, USA).

An initial exploratory analysis was conducted to examine the fitting of the quantitative data to the normal distribution using the Shapiro–Wilk test [19]. To compare the differences between the techniques over time, we formulated a Friedman two-way analysis by ranks, dividing the experimental period into 5 time periods. The periods were chosen to reflect the different phases of wound healing:

- Time period A (1st–8th po.d.), when inflammation, debridement and proliferation take place;
- Time period Β (9th–21st po.d.), when proliferation takes place;
- Time period C (22nd–63rd po.d.), when the early stage of maturation takes place;
- Time period D (64th–180th po.d.), when the median stage of maturation takes place;
- Time period E (181st–365th po.d.), when the late stage of maturation takes place.

In order to obtain representative (total) scores for the cosmetic, clinical, ultrasonographic and histological evaluations, for each time period, separate scores for some of the parameters in each category were added.

For cosmetic examinations, intraobserver variability was first examined with the Mann–Whitney U test. Afterwards, the total score was calculated by averaging the separate scores given by the two observers.

For total clinical evaluations, the scores for skin thickening, scar width, hyperpigmentation and inflammation were added. The values for skin thickening and scar width, before being added, were transformed to a 4-scale score (0–3), based on the 25th, 50th and 75th percentiles of the distribution of the total values.

For the ultrasonographic evaluations, four different tomographic sections (a, b, c and d) were examined at four sites along the scar of each incision, “a” being proximal and “d” being distal to the hip joint. The wound area was calculated for each tomographic plan. The differences between the volumes of the tomographic sections were tested with the Wilcoxon signed rank test, due to salient violation of the normal distribution. Furthermore, the mean u/s wound area of each wound (derived from the four volume values taken for each wound) was transformed to a 4-scale score (0–3), based on the 25th, 50th and 75th percentiles of the distribution of the total values.

For total histological evaluations, the scores for oedema, inflammatory reaction, thickness of the epidermis at the area of wound healing and scar width were added. The values for the thickness of the epidermis at the area of wound healing, the epithelial gap and scar width, before summation, were transformed to a 4-scale score (0–3), based on the 25th, 50th and 75th percentiles of the distribution of the total values. Tissue necrosis and epithelial gap were not included, as there was no sign of them in any of the samples, and no further analyses were conducted. Collagen deposition, fibroblast presence and angiogenesis were also not included in the summation, as they did not differ between the techniques.

Furthermore, in order to determine whether there was any correlation between u/s estimated mean wound areas and various histological and clinical parameters, Spearman’s rank order correlation coefficient was used.

Finally, to compare the techniques over time, the total representative score for each technique was evaluated for each time period by adding the cosmetic, clinical, ultrasonographic and histological total scores.

## 3. Results

### 3.1. Technique Duration

The times required for each skin-closure technique are shown in Table 1. A comparison showed that they significantly differed (*p* < 0.001), with intradermal B requiring almost 2 min more than intradermal C.

### 3.2. Cosmetic Evaluation

There was no statistically significant difference between the scores of each of the two assessors during the evaluation of the cosmetic appearance of the wounds (*p* = 0.91). Moreover, no statistical difference was observed between the median scores of each of the two assessors in accordance with the technique employed. With regard to the intradermal B technique, the median score for the first assessor was 1.5 (1–2) and that for the second was 2.0 (1–2), whilst, for intradermal C, the median scores were 1.5 (1–2) and 1.0 (1–2), respectively. The scores for the total cosmetic evaluations (average scores) for each time period are presented in Table 2. Based on the results, in our sample, we could not find a statistical difference between the cosmetic evaluations of the techniques for any time period.

### 3.3. Clinical Evaluation

#### 3.3.1. Skin Thickness

The median skin thickening (mm) in each period are presented in Table 3. Following the results of the analysis, no statistically significant differences were observed between the two techniques for any time period. With the intradermal B technique, skin thickening was more pronounced in the knot areas; however, these areas were not included in the evaluations. Swelling appeared from the 5th po.d., and in some animals remained as mild swelling until the 42nd po.d.

#### 3.3.2. Erythema

Erythema was more intense 24 h after suturing and usually lasted 3 to 6 days postsurgery for both techniques. In period B, it remained in two animals that underwent intradermal C, in one until day 10 and in the other until day 18. No erythema was observed afterwards. In period A, the median erythema (in mm) was 0.3 (0.0–0.7, max. 3.8) for intradermal B and 0.3 (0.0–0.6, max. 3.9) for intradermal C, with no significant differences between the techniques.

#### 3.3.3. Scar Width

The median scar width (in mm) for each period are presented in Table 3. The last measurement of scar width, taken one year postoperatively, showed a thinner scar for intradermal B than intradermal C. In the present sample, we could not observe statistically significant differences between the techniques for any time period.

#### 3.3.4. Inflammation and Abscessation

**Figure 4:**
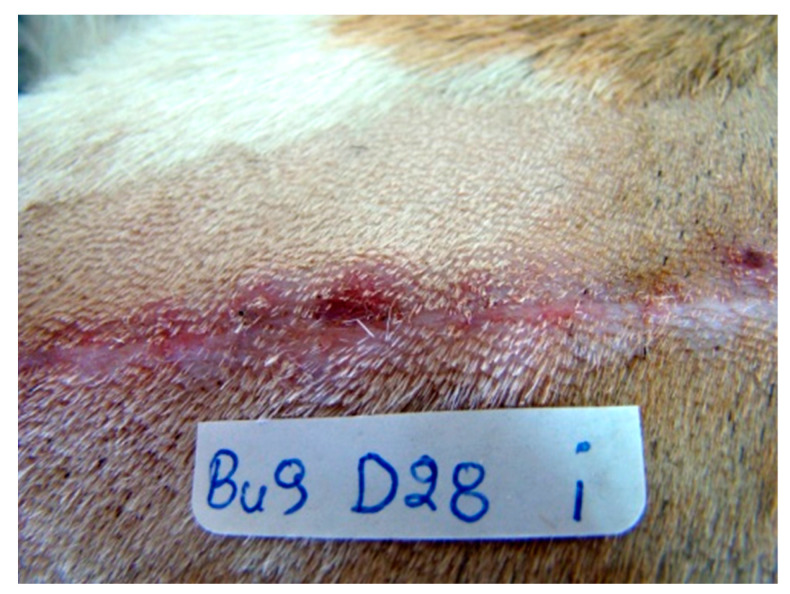
A case of a microabscess in animal No. 9 on the 28th po.d. at the wound sutured with intradermal B.

Inflammation was observed in four animals, and in all but one wound was evaluated with a score of 1, i.e., mild inflammation. Inflammation was observed in animal No. 5 on the first two po. days in the wound closed with intradermal B. In animal No. 9, it was observed from the 21st to the 35th po.d. in the wound closed with intradermal B. In animal No. 10 it was observed from the 18th to the 77th po.d. at the wound closed with intradermal B. With intradermal C, there was only one case of mild inflammation, in animal No. 7, on the 16th po.d. In only one case, a microabscess was noticed in animal No. 9 on the 28th po.d. at the wound sutured with intradermal B (Figure 4). There was no statistically significant difference between the numbers of inflammation incidents observed for intradermal B (15 cases in 3 animals) and intradermal C (1 case in 1 animal).

#### 3.3.5. Exudate

No exudate was observed in any incision of any animal.

#### 3.3.6. Comedones

Comedones were observed only on two occasions (on the 35th and 42nd po.d.) in one animal with an incision closed by intradermal B. However, no significant difference was found between the techniques.

#### 3.3.7. Hyperpigmentation

Hyperpigmentation was absent or mild with both techniques, and when observed (usually after day 28) remained for a short period of time. The median wound hyperpigmentation score for each period is presented in Table 3. No significant differences were detected between the techniques.

#### 3.3.8. Suture Loss and Wound Dehiscence

No suture loss or wound dehiscence was observed in any incision of any animal.

#### 3.3.9. Total Clinical Evaluation

The scores for the total clinical evaluations for each time period are presented in Table 3. No significant differences were detected between the techniques for any time period.

### 3.4. Ultrasonographic Evaluation

**Figure 5:**
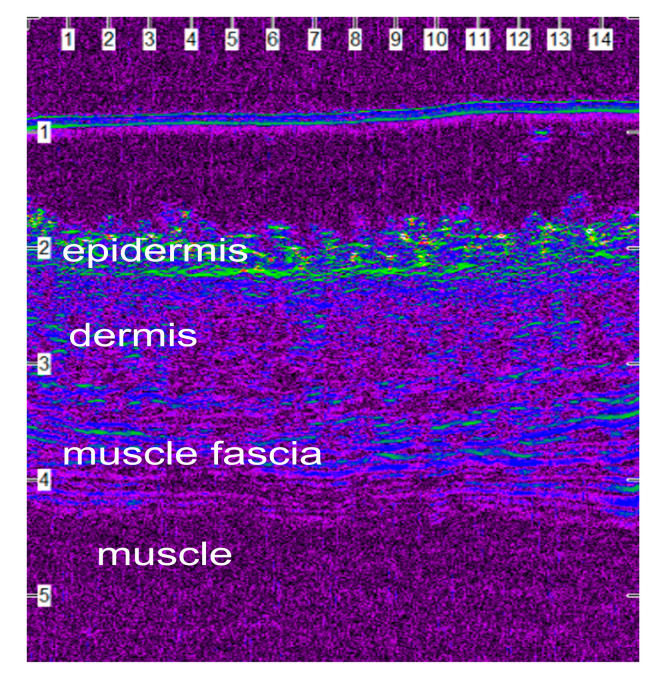
Ultrasonographic image of the normal canine skin of the lateral thigh. The image is compressed laterally to facilitate viewing of the wound area (scale in mm).

Before skin incision, in all of the ultrasound scans the epidermis was clearly visible as a hyperechoic linear layer. The dermis had a granular echotexture that appeared to become more linear in the deeper parts. Subcutaneous tissue was recognized at a greater depth, as a thicker layer characterized by an inhomogeneous hypoechoic or nonechogenic pattern (Figure 5).

**Figure 6:**
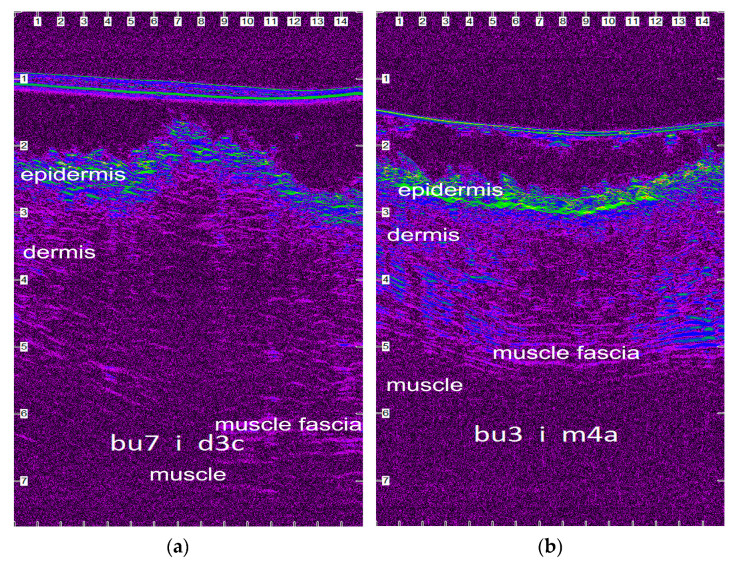
Ultrasonographic images of the wound areas sutured with intradermal B 3 days (**a**) and 4 months (**b**) postoperatively. The images are compressed laterally to facilitate viewing (scale in mm).

After wound closure, the ultrasonographic image of the skin at the wound area differed from the adjacent normal one. The shape of the epidermis was deformed, creating a cone that protruded 1–2 mm. The area of the dermis at the incision site was enlarged and hypoechogenic compared to the normal adjacent one. Specifically, the wound area was ultrasonographically (u/s) defined dorsally by the two echogenic, uplifted epidermal edges, laterally by the two echogenic and thickened dermal edges, and ventrally by the anechoic oedematous subcutaneous tissue and muscle fascia (Figure 6a). As wound healing proceeded, the wound area size and tissue oedema diminished, and the progressive collagen deposition altered echo intensity, making wound boundaries more complex but not indistinguishable (Figure 6b).

After the 15th po.d., the ultrasonographically estimated wound area was reduced by half with both techniques. From the 40th to the 120th po.d., the wound area continued to reduce in size with both techniques, whilst the epidermis at the wound area lost its conical shape and became flat, with a density similar to that of the adjacent normal skin. The wound area was depicted more clearly with intradermal B compared to intradermal C, as with the last technique the wound area was almost isoechoic to the normal adjacent skin. After the 150th po.d., the newly formed mature dermis at the wound area became almost isoechoic to the normal adjacent skin in all incisions with both techniques. The wound area was calculated for each tomographic plan. As the ultrasonographically estimated wound areas differed significantly between the four different tomography planes of each incision, the mean areas were calculated and used for further evaluations. The median u/s estimated wound area for each time period are presented in Table 4. The differences between the techniques were statistically significant in periods A, B and C (*p* = 0.029, 0.025 and 0.011, respectively), but not afterwards.

### 3.5. Histological Evaluation

#### 3.5.1. Necrosis

No necrosis was observed in any tissue examined.

#### 3.5.2. Epithelial Gap

No epithelial gap was observed in any tissue examined.

#### 3.5.3. Oedema

In period A, oedema was observed in 5/10 samples (with score 1) with both intradermal B and C. In periods B, C, D and E, no oedema was observed with any technique.

#### 3.5.4. Inflammation

The inflammatory reaction scores are presented in Table 5. No statistically significant difference was observed between the techniques for any period.

#### 3.5.5. Presence of Suture Material and Tissue Reaction

**Figure 7:**
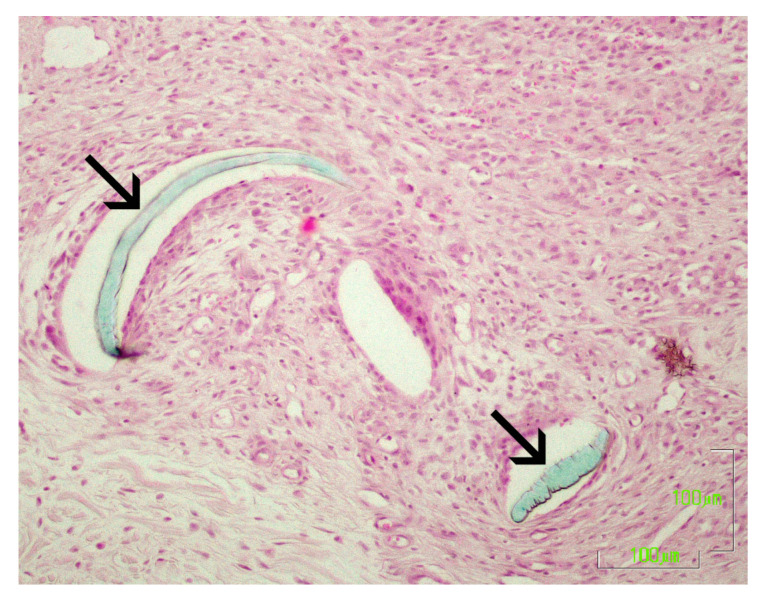
Remnants of suture material (arrows) at wound area, after removal of polypropylene suture (intradermal C), on the 14th po.d.

The presence of suture material or its initial position was identified in 24 samples with poliglecaprone 25 and 6 samples with polypropylene. From the 14th po.d. onwards, polypropylene was not found, as the suture material had been removed on the 10th po.d., while the polypropylene’s initial position was identified in two of the samples. Remnants of suture material at the wound area were found in one case after suture removal (Figure 7). Poliglecaprone was not found on the 180th po.d., due to its absorption within 119 days postimplantation.

**Figure 8:**
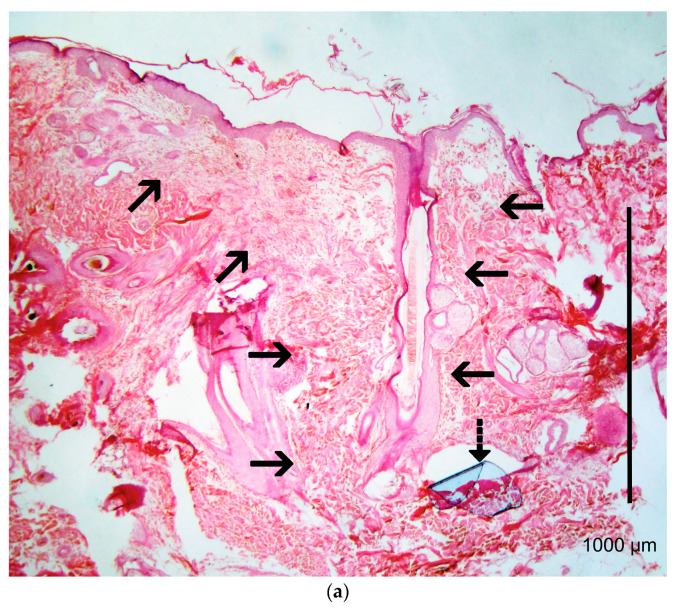

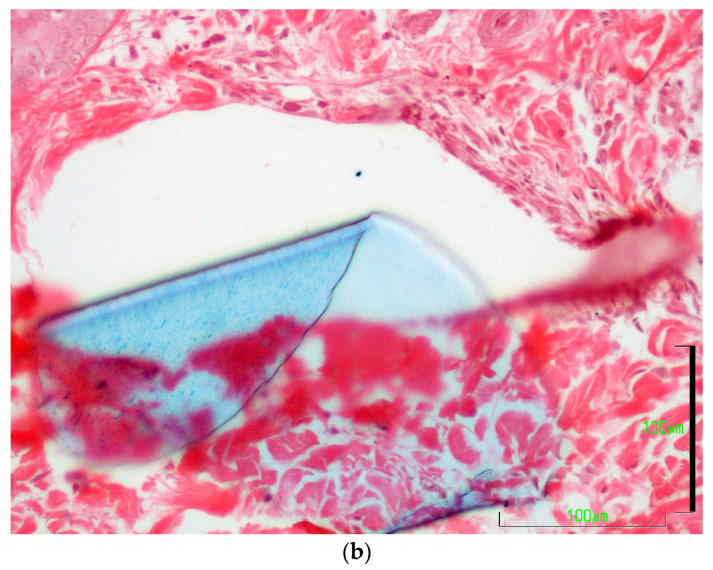
(**a**) Wound area at the site of incision (black arrows) on the 7th po.d. after suturing with polypropylene (blue film partially relocated, dotted arrow). (**b**) Close-up of polypropylene suture showing minimal tissue reaction composed of macrophages and fibroblasts around suture material.

**Figure 9:**
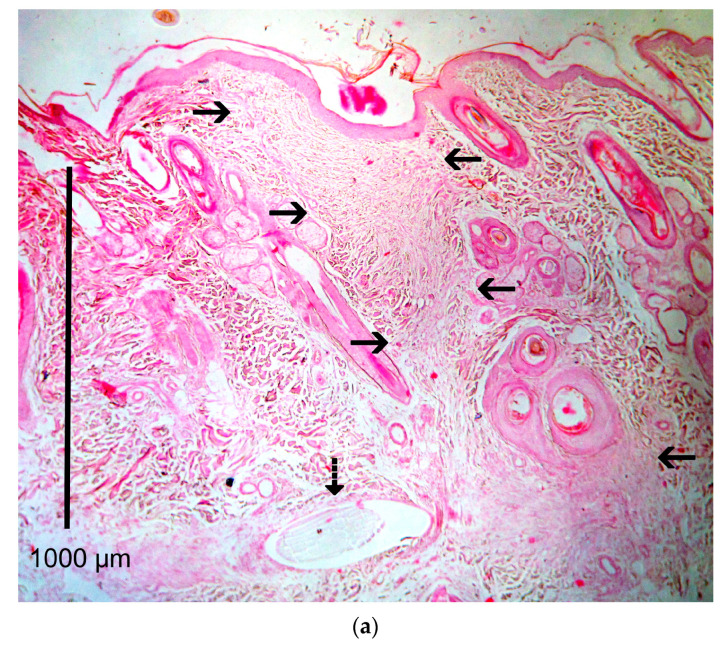

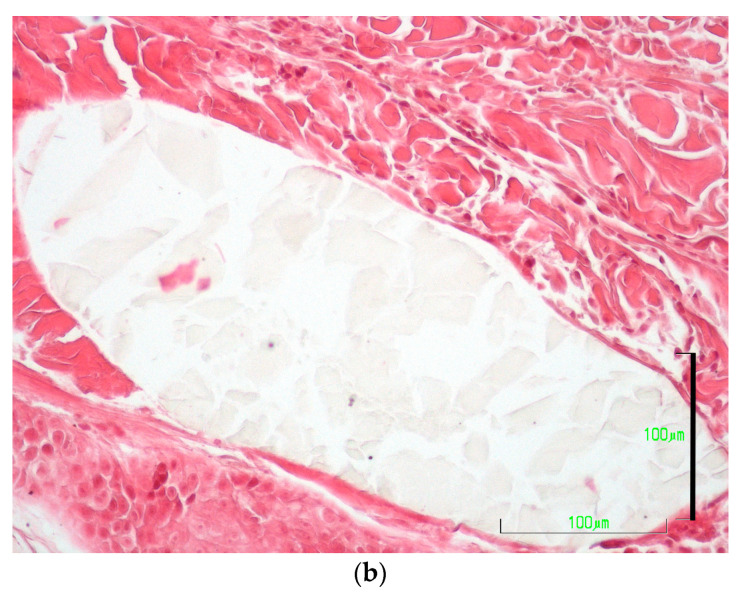
(**a**) Wound area (between arrows) at the site of the incision on the 14th po.d. after suturing with poliglecaprone 25 (dotted arrow). (**b**) Close-up of poliglecaprone 25 suture showing minimal tissue reaction observed around suture material (scale bar: 100 μm).

On the 7th po.d., the tissue reaction around the suture material consisted of macrophages and fibroblasts, and it was evaluated as minimal as regards intradermal C, where polypropylene was used (Figure 8a,b). As regards intradermal B, on the 7th, 14th and 28th po.d., the tissue reaction around the suture material was minimal to moderate, and it consisted of macrophages and fibroblasts. In some cases, the suture material was only surrounded by normal skin collagen fibers and not by cells; in other cases, it was surrounded by a small number of macrophages arranged in one or two layers, or it was surrounded by more than one layer of inflammatory cells, which layers were surrounded by fibroblasts and circular collagen fibers (Figure 9a,b).

From the 180th po.d. onwards, the area that the suture material had passed through was not detected in any tissue sample.

Tissue reaction around the suture material as regards intradermal B was evaluated for periods A, B and C (the suture was absorbed 90–110 days after implantation) and as regards intradermal C in period A (the suture was removed 10 days after implantation).

In period A, the scores for the tissue reactions around the suture materials as regards intradermal B were 1 in four samples, 2 in four samples and 3 in one sample; in period B, the scores were 1 in three samples, 2 in four samples and 3 in one sample; and in period C, the scores were 1 in three samples, 2 in one sample and 3 in two samples. As regards intradermal C, in period A the score for tissue reaction around the suture material was 1 in four samples. Due to the small number of samples, no statistical analysis was performed.

#### 3.5.6. Epithelial Thickness

The median thickness values for the epidermis at the area of wound healing (the number of times by which the thickness was greater than that of the adjacent healthy epidermis) are presented in Table 5. No significant differences were observed between the techniques.

#### 3.5.7. Scar Width

The histologically estimated median scar widths (in mm) for each technique for each period are presented in Table 5. No statistically significant differences were observed between the techniques.

#### 3.5.8. Collagen Deposition, Fibroblast Presence and Angiogenesis

The collagen deposition scores, fibroblast presence scores and angiogenesis scores showed no statistically significant differences between the techniques for any period.

#### 3.5.9. Total Histological Evaluation

The total scores for the histological evaluations for each time period are presented in Table 5. For all periods, no significant differences were observed between the techniques.

#### 3.5.10. Total Evaluation

**Figure 10:**
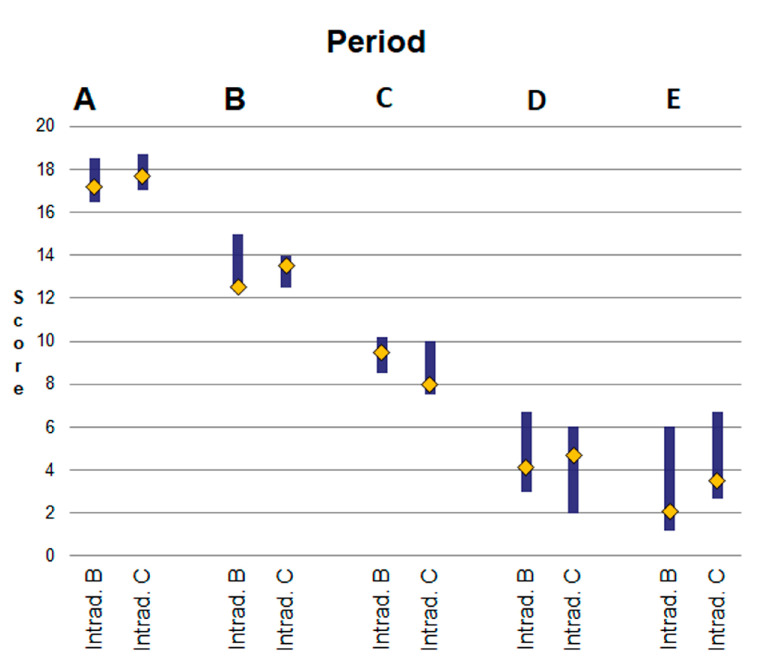
Medians (yellow squares) and interquartile ranges (columns) of total evaluation scores for each technique for each period. No significant differences were observed between the techniques for any time period.

For period A, the median total evaluation score was 17.2 (16.5–18.5) for the intradermal B technique and 17.7 (17–18.7) for intradermal C. For period B, the median total evaluation score was 12.5 (12.2–15) for intradermal B and 13.5 (12.5–14) for intradermal C. For period C, the median total evaluation score was 9.5 (8.5–10.2) for intradermal B and 8.0 (7.5–10) for intradermal C. For period D, the median total evaluation score was 4.1 (3.0–6.7) for intradermal B and 4.7 (2.0–6.0) for intradermal C. For period E, the median total evaluation score was 2.1 (1.2–6) for intradermal B and 3.5 (2.7–6.7) for intradermal C (Figure 10).

For all periods, the comparisons revealed no significant differences between the techniques.

### 3.6. Correlations

#### 3.6.1. Correlation between u/s Estimated Wound Area and Clinically Evaluated Skin Thickening

Spearman’s correlation was implemented to test the association between the u/s estimated wound area and the clinically evaluated skin thickening. The results showed that there was a statistically significant positive correlation between the two parameters (r = 0.682, *p* < 0.001), i.e., the larger the u/s estimated wound area, the greater the skin thickening.

#### 3.6.2. Correlation between u/s Estimated Wound Area and Histologically Estimated Scar Width

Spearman’s correlation for the u/s estimated wound area at the biopsy site and the histologically estimated scar width in the same area showed that there was a statistically significant positive correlation between the two parameters (r = 0.342, *p* < 0.001), i.e., the larger the u/s estimated wound area, the greater the scar width.

## 4. Discussion

Various suturing techniques have been described in the literature for their specific benefits with respect to wound closure. It is generally assumed that the intradermal suture pattern has superior cosmetic results, mainly because the epidermis is not penetrated [20] such that inflammation is minimal, whilst a fine approximation of wound edges can also be achieved, resulting in minimal scarring [21]. Intradermal skin closure may be achieved using either a continuous absorbable or a nonabsorbable monofilament suture material [1,22]. Polypropylene is a nonabsorbable material with minimal tissue drag that was favorably compared to absorbable monofilament materials for intradermal closures in human surgery [23]. However, only a few studies have aimed to assess the intradermal suture pattern objectively, under controlled experimental conditions [4,5,6]. Further, there has been no study on the impact of the use of nonabsorbable sutures without burying of the knots on wound closure with an intradermal suture pattern in dogs.

Among the intradermal suture techniques performed in this study, the pattern with clips was easier to perform compared to the pattern with burying of the knot and required significantly less time than the second. The tying and correct burying of knots into the subcutaneous tissue is more technically demanding and time-consuming than the stabilization of suture materials with clips, but not a reason for an experienced surgeon to avoid this technique, especially in areas where proper protection of the wound site with dressings is not feasible. Concerning suture materials, the handling of 4/0 poliglecaprone 25 is easier than that of 4/0 polypropylene, as the former is more pliable.

### 4.1. Cosmetic Evaluation

In general, scar appearance for both techniques was cosmetically evaluated by the assessors, with good ratings. Although the cosmetic evaluations of the wound areas gradually improved for both techniques, there were better scores until the 180th day for intradermal C, probably because the suture material had been removed from the wound sites, such that the mechanical irritation of the skin was less. The opposite was found at the end of the experiment, i.e., 365 days postoperatively, probably because the longer stabilization of the wound edges with intradermal B aided the prevention of scar widening and resulted in thinner scars. However, these differences were not confirmed statistically.

### 4.2. Clinical Evaluation

Wound repair by first intention was achieved normally with both techniques, with no severe complications. Skin thickening, erythema, scar width, abscessation, hyperpigmentation, wound and dehiscence were the parameters assessed in the total clinical evaluation.

On clinical evaluation, thickening of the skin at the incision line was observed with both suturing techniques from day 1. In period A (1st–8th po.d.), as the wound margins were held tight throughout the whole length of the incision by the sutures, the tissue swelling at the wound area was mild. In period B (9th–21st po.d.), as the wound healing progressed without complications, skin thickening decreased for both techniques. Almost from the beginning of this period (10th day), only the poliglecaprone 25 suture was still inside the wound area, since the polypropylene suture had been removed. Greater skin thickening at the wound areas was observed with intradermal B, probably due to the presence of suture material in the wounds at that period, without any significant differences observed between them. It must be noted that, with intradermal B, in the areas where the knots were buried, there was more pronounced swelling, which remained for longer than in the rest of the incisions. In period C (22nd–63rd po.d.), skin thickness at wound areas for both techniques decreased further, without significant differences between the techniques. Afterwards, wound skin thickness decreased to prewounding values. Gouletsou et al. [6] observed that skin thickening at wound areas treated with the intradermal technique with burying of the knots and with the use of 4/0 absorbable monofilament suture materials was large on the 1st po.d. and then decreased gradually until the 20th po.d. In contrast, when the intradermal suture pattern with burying of the knots was performed using an absorbable monofilament suture material with a larger diameter (3/0), skin thickening increased from the 1st until the 12th po.d. and then decreased gradually. Papazoglou et al. [4] found that incisional swelling in cats tended to be longer-lasting and more prevalent in incisions sutured with absorbable sutures than those sutured with polypropylene.

Erythema was also observed in the wound areas. From the 1st until the 8th po.d., it was mild for both intradermal B and intradermal C. From the 9th until the 18th po.d., it was only observed in two wounds sutured with intradermal C. Although erythema is expected during the inflammation stage of the healing process, in this study, erythema was mild with both techniques. Gouletsou et al. [5,6] also observed erythema at wound areas for all incisions sutured with intradermal patterns; however, it was more intensive and lasted longer, i.e., until the 49th po.d. Gouletsou et al. [5,6] did not administer any antibiotics postoperatively, as was performed in the present study, and this may have caused a rather prolonged inflammation phase that aggravated erythema. Sylvestre et al. [3] also noticed that the intradermal pattern induced erythema in dogs in the first po. days, which declined on days 10–14.

A significant clinical parameter of the skin healing process is the scar that forms on the skin at the wound area. In the present study, on the first po. days, the scars were thin and covered with eschars (scabs). After the eschars had fallen off, the regions of the scars were distinguished due to the different textures and because they were colourless and hairless. The scars remained thin with both techniques until the 21st po.d., while, from the 24th until the 63rd po.d., scar width slightly decreased with intradermal B and slightly increased with intradermal C. Probably, the two-layer closure of the incisions, with subcutaneous and intradermal suturing, improved scar width with intradermal C during this period, when intradermal sutures had been removed. With both techniques, scar width reduced gradually afterwards. At the final measurement, one year postoperatively, it was slightly smaller for intradermal B; however, the differences between the two techniques were insignificant for the whole study period.

Similar findings were observed by Vipond and Higgins [23], who examined skin healing in humans undergoing abdominal surgery randomly allocated to have their skin wounds closed by either polypropylene (Prolene) or polydioxanone (PDS) by the subcuticular method with burying of the knots. They found no differences in scar widths between the techniques three months after suturing. Gouletsou et al. [5] compared the scar widths between intradermal techniques with burying of the knots (without subcutaneous suturing) using absorbable monofilament suture materials of different diameters (4/0 poliglecaprone 25 and 3/0 poliglecaprone 25) in dogs. The narrowest scar was observed with the intradermal technique using a 4/0 suture. Gouletsou et al. [6] also compared scar width with respect to intradermal techniques with burying of the knots using absorbable monofilament suture materials of different absorption times (4/0 poliglecaprone 25 absorbing at 100 days and 4/0 polyglytone 6211 absorbing at 56 days) in dogs and found that early absorption did not have a beneficial effect on scar width.

In the present study, a few cases of mild inflammation were observed with both techniques, whilst exudate discharge or wound dehiscence was not observed for any of them. Most of the cases occurred with intradermal B, and only one case was observed with intradermal C. Inflammation was usually observed in the first po. days and lasted 1–4 days, except for one wound sutured with intradermal B, where the mild signs of inflammation lasted two months. Gouletsou et al. [5,6] observed more severe cases of inflammation and microabscess formation in intradermal suture patterns until the 35th po.d., which, in four cases were present for 3–5 days, and in three cases for 15–30 days. This can probably be explained by the fact that no antibacterial drugs were administered postoperatively to these animals. In contrast, the amoxicillin/clavulanic acid administered to the animals during the initial 10 po. days in the present study might have contributed to the low incidence of inflammation. It can be concluded that, even in noncontaminated clear surgical wounds, po. administration of antibacterial agents may reduce wound inflammation and prevent microabscess formation, which otherwise might occur.

In the present study, comedones were observed on only two occasions, 35 and 42 days postoperatively, in the same incision closed by intradermal B. Comedones have been connected with post-suturing scars in only three studies, i.e., Webster et al. [24], who reported comedones after suturing eyelid skin with catgut, and Gouletsou et al. [5,6], who, 20 to 90 days postoperatively, observed comedones in almost all wounds sutured with intradermal patterns. Smeak [1] suggested that epithelial cysts may form after traumatizing the *stratum basale* of the epidermis or the hair follicles during the passage of needle and suture, and Gouletsou et al. [5,6] assumed that this could be an explanation for the presence of comedones at the scars observed in their study. However, since, in the present study, only minimal comedones were observed, even though the experiments were performed in the same environment by the same surgeon, it should be suggested that, with some probability, antibiotic administration and elimination of inflammation might explain their absence.

Alteration of scar colour was another characteristic of skin healing in the present study. In the majority of animals that underwent both techniques, from the 35th until the 180th po.d., the scar acquired a darker colour than that of the adjacent skin. Then, until the 365th po.d., the colour of the scar was slightly darker with intradermal C than with intradermal B, without this difference being significant. According to Swaim and Henderson [22] and Hosgood [25], the first signs of pigment deposition in the skin become visible 1–2 weeks postoperatively, although the maximum concentration of melanocytes is observed several months later. Melanogenesis depends on both exogenous and endogenous agents, such as alpha-melanochrostikotropine (a-MSH) and adrenocorticotrophic (ACTH) hormone, vitamin D3, interleukins or other cytokines, which influence the process of melanin production from melanocytes [26,27]. The change in scar colour was observed in approximately the 4th po. week, probably due to the action of produced cytokines which stimulated the production of melanin [28,29]. In the present study, the pigmentation was mild and of shorter duration than that observed by Gouletsou et al. [5,6]. Gouletsou et al. [5,6] observed that scars became darker than adjacent normal skin from the 28th po.d. until the 180th po.d., and, in some cases, until the 365th po.d., in most of the wounds sutured with an intradermal suture pattern or a simple interrupted pattern; however, no statistically significant differences were observed between the techniques.

In the present study, in order to gain an overall view of the total clinical evaluation of each technique, some of the scores for clinical parameters were summed and total clinical scores for each of the five periods were calculated. As was expected, total clinical scores improved over time with both techniques, being the same for periods A–D for both techniques, and slightly better for intradermal B in period E, without the difference being significant.

### 4.3. Ultrasonographic Evaluation

#### 4.3.1. Ultrasonographic Findings

To evaluate ultrasonographically the skin healing process over time, B-mode ultrasonography was used and wound area was estimated. The surgical closure of the skin edges created a compartment that was filled with a serohaemorrhagic exudate derived from blood plasma as a product of the acute inflammation caused by surgery that was ultrasonographically recorded as a hypoechoic to an anechoic small region. On the first po. days, the lower limits of the wound areas were difficult to identify due to the similar echogenicity of the subcutaneous tissues to the oedematous wound areas. As wound healing progressed, wound area size and tissue oedema diminished, and progressive collagen deposition altered echo intensity, making wound boundaries more complex but not indistinguishable. Generally, from the 9th until the 365th po.d., smaller wound areas were consistently recorded for intradermal C, probably because there were no suture materials inside the wounds to induce tissue reactions. In contrast, with the intradermal B technique, the concurrent tissue reaction to the suture materials present in the wound areas, up to the 119th po.d., contributed to the larger wound areas. It should also be mentioned that, on the 365th po.d., wound areas could not be detected in a few segments of some wounds treated with both techniques, suggesting complete skin repair.

#### 4.3.2. Correlations between Ultrasonographic Findings and Clinical and Histological Findings

Skin ultrasonography is a new technique used in studies on canine skin, and only a few relevant studies have been published so far—one on the evaluation of the use of an aliamide-containing gel in the treatment of skin wounds [30] and another on the evaluation of skin hydration status and pathological modifications of skin hydration in dogs [31].

The normal appearance of the skin of Beagles as observed with high-frequency ultrasound has been reported by Mantis et al. [32], in whose study a correlation with histological appearance was found. The present study is the first to use high-frequency ultrasonography to observe the way that skin heals after the use of skin-closure techniques that aim at healing by first intention. Furthermore, in order to determine whether high-frequency ultrasonography depicts the real ultrastructure of skin during first-intention healing, some correlations have been examined.

The test of the correlation between u/s estimated wound area and clinically evaluated skin thickening showed that there was a statistically significant positive correlation between the two parameters, i.e., the larger the u/s estimated wound area, the greater the skin thickening measured clinically with a skin caliper. Based on this finding, it is feasible to calculate skin thickness at a surgically closed incision without the need to use a skin caliper, which cannot be used in small areas, in areas where the skin cannot be folded or when the procedure is painful.

The test of the correlation between u/s estimated wound area at the biopsy site and histologically estimated scar width in the same area showed that there was a statistically significant positive correlation between the two parameters. Therefore, the larger the u/s estimated wound area, the greater the scar width calculated histologically. This correlation existed throughout the whole experimental period, which was much longer than the that of the first po. days when inflammation was present. After the inflammation subsided, ultrasonography detected differences in collagen deposition, since collagen bundles in wound areas are finer than those in nearby normal skin. However, on the 365th po.d., scar area had not been detected in all ultrasonography scans, probably because collagen density at the wound area did not differ significantly from that in the nearby area.

Based on all the aforementioned results, it can be concluded that high-frequency ultrasonography can be used to evaluate first-intention healing of canine skin. Ultrasonographic findings correlated very well with the clinical and histological findings and depicted the real ultrastructure of the skin during first-intention healing.

### 4.4. Histological Evaluation

Histological examination gives detailed information on the healing process of traumatized skin that may not be revealed by any other method. It shows the causative agent of skin thickening, the presence of inflammation and the types of cells involved in it, the presence of suture material and the tissue reaction to it, the epithelial gap and thickness at the wound area, scar width, and the way tissues heal by means of collagen production, the presence of fibroblasts and angiogenesis. It is also important that all of the above parameters are countable, and, as a result, statistical significance may be determined. However, in clinical settings, where animals or humans are used, performing multiple skin biopsies during healing is very difficult or even unfeasible. This is the reason why only a few studies providing histological information on skin healing can be found and why even fewer studies have been performed on canine skin. The most detailed studies on canine skin healing were conducted by Gouletsou et al. [5,6], who investigated skin healing by clinical examination and histopathology, after suturing with an intradermal suture pattern and using sutures with different diameters and of different materials.

In this study, oedema was a constant finding on the 7th po.d. It seems that oedema is an expected finding during the first week of healing and is not affected by the technique of skin closure. However, it is connected to the presence of inflammation in the wound area, and, in such cases, may last even one month postsurgery. Gouletsou et al. [5] also found oedema in almost all wounds examined on the 7th po.d. with all the techniques performed, in half of the samples on the 14th po.d., and in a few samples on the 28th po.d. In their study, all the cases of oedema after the second po. week were found in samples where inflammation was present [5]. Since they gave no antimicrobial drugs, inflammation and oedema were more common and lasted for longer periods of time [4,5]. Papazoglou et al. [4] evaluated oedema in six cats subjected to skin suturing with an intradermal suture pattern using either a copolymer of glycolide, caprolactone and trimethylene carbonate or a polypropylene suture with clips. In their study, oedema was present in all the incisions sutured with the first suture on the 2nd po.d. but subsided on the 7th po.d. In contrast, swelling of incisions was present in three cats sutured with polypropylene on the 2nd po.d. but subsided in all cats on the 7th po.d.

In the present study, no significant difference in infiltration with leukocytes was observed between the techniques, indicating that skin healing was not affected by the presence of suture materials and that the presence of leukocytes was probably a consequence of the normal healing process. Gouletsou et al. [5,6] also observed that medium-to-high numbers of neutrophils and macrophages infiltrated wound areas during the first 14 days postoperatively when the wounds were sutured with intradermal suture patterns with burying of the knots. However, in their study, more cases of severe inflammation (perivascular, nodular or diffuse) were detected, probably because no antibiotics were administered postoperatively. Papazoglou et al. [4] evaluated inflammation in six cats following suturing of the skin with intradermal suture patterns using copolymer of glycolide, caprolactone and trimethylene carbonate sutures or polypropylene sutures with clips and noticed that cellular reactions were moderate to severe in most incisions, regardless of the closure technique. This might be due to the trauma produced by the placement of the needle and to the reaction to the suture material.

In the present study, in general, during the first po. days, with both techniques, the epidermal thickness at the area of wound healing increased compared to the adjacent normal skin. For both techniques, in period A it was 2.5 times the thickness of the normal one, decreased to twice the normal thickness in period B and returned to normal from period D onwards. Increased epidermal thickness was not restricted to the newly formed epithelium but was also extended to the intact wound edges. The intense proliferation of cells at wound borders replaced losses due to the continued movement of epidermic cells to the epidermal deficit [33]. Papazoglou et al. [4] observed that on the 9th po.d. thickness was greater in four out of six sections which included glycolide, caprolactone and trimethylene carbonate, whilst it was normal in all sections that included polypropylene. In the present study, epithelial thickness at incisions sutured with polypropylene returned to normal one month postoperatively; however, the method of measuring epithelial thickness used in this study differs from that of Papazoglou et al. [4]. Furthermore, species differences may contribute to this discrepancy. Gouletsou et al. [5] also observed two- to three-fold increases in epithelial thickness on the 7th po.d. with the intradermal suture techniques used, and on some occasions observed a five-fold increase. The increase in their study subsided on the 28th po.d. and was smaller at incisions closed by the intradermal technique with burying of the knots performed with 4/0 poliglecaprone 25.

In the present study, an epithelial gap was not observed in any sample on the 7th po.d. or afterwards. Gouletsou et al. [5] observed that epithelial bridging was delayed more and that half of the samples for the techniques used presented epithelial gaps on the 7th po.d. Perhaps, in the present study, the use of antibiotics postoperatively contributed to the faster epithelialization observed for both techniques. Pope [34] also found that epithelialization was complete in the first po. days after surgical skin closure. Kirpensteijn et al. [2], who checked the epithelial bridging after suturing skin incisions with intradermal sutures with either poliglecaprone 25 or polyglactin 910, also observed that all the wounds had been epithelialized on the 7th po.d.; however, they gave no information about any antibiotic administration. Papazoglou et al. [4] noticed that epithelial bridging was completed at the 9th po.d. in all incisions closed with polypropylene but in only one out of six incisions closed using the other technique.

For all time periods, no significant differences were revealed between the techniques in terms of histologically evaluated scar width, and scar width was found to become narrower over time. The only other study of histologically evaluated scar width is that of Gouletsou et al. [5], who observed that scar width was greater in incisions sutured with simple interrupted stitches in comparison to those sutured with intradermal suture patterns. Their findings were constant for the whole study period, which was three years postoperatively. In that study, the intradermal suture pattern with burying of the knots produced a scar of approximately 0.7 mm in width, which is slightly larger than the values found in the present study, i.e., 0.45–0.56 mm. This difference may be partly the result of antibiotic administration and the less intense inflammation observed in the present study.

Generally, tissue reaction around polypropylene was minimal and composed of a few cell layers containing macrophages and fibroblasts. With intradermal B, in the first po. month, tissue reaction was evaluated as minimal to mild, containing macrophages and fibroblasts. From the 180th po.d. onwards, the area where the suture material had been implanted was impossible to detect in any tissue sample. It has been reported that after full absorption of the suture material, the area where it is located is characterized by the presence of macrophages surrounded by fibroblasts [35,36,37]. However, in the present study, accumulations of macrophages were only observed around hair and debris, probably as a result of traumatic furunculosis.

The collagen-deposition scores, fibroblast-presence scores and angiogenesis scores did not differ among the techniques for any period of the study. Van Winkle et al. [38], who measured biochemically the collagen quantities in skin wounds closed with different suture materials, observed that collagen production was not affected by the type of suture material used. Gouletsou et al. [5,6] also found no difference in the number of fibroblasts, collagen production and angiogenesis between the techniques used. Papazoglou et al. [4], when they evaluated wound vascularity and collagen contents in six cats after suturing their skin with an intradermal suture pattern using either a copolymer of glycolide, caprolactone and trimethylene carbonate or a polypropylene suture with clips, observed no significant differences between the two techniques. However, it is possible that the scoring system for collagen production that was used in the present study, after being established in many similar studies, would not be appropriate to estimate subtle differences in collagen production during wound healing by first intention.

The total histological evaluation for each time period was recorded after summing up the individual scores for oedema, inflammation, tissue reaction around the suture material, thickness of the epidermis at the wound area and scar width and revealed no differences between the techniques.

### 4.5. Total Scores for Each Technique

After summing the scores for the cosmetic, clinical, ultrasonographic and histological evaluations for each time period, the total score for each technique was calculated in an attempt to estimate the overall outcome of each technique over time, even though the conclusions should be treated with some caution. Thus, in periods A and B, intradermal B presented better total score values than intradermal C. However, in period C, intradermal C had better total score values than intradermal B, because, one month postoperatively, the former caused mechanical irritation of the healing area and foreign body inflammatory reaction. Gouletsou et al. [5,6] also found that with intradermal suture patterns with burying of the knots, the good cosmetic outcomes observed during the first po. period deteriorated during the 4th-8th po. weeks and improved again afterwards, when the suture material was absorbed. The use of an early-absorbed suture, such as polyglytone 6211 [6], did not improve the cosmetic outcome of wound healing during this period, since it was absorbed later, on the 56th po.d. In period D, intradermal B presented a better total score than intradermal C, because inflammation, being the result of either the healing process or foreign-body reaction, had subsided, as the poliglecaprone 25 suture material had been absorbed two months previously and probably because the skin edges were supported for a longer period of time, preventing expansion of the scar. Finally, in period E, intradermal B still had a higher total score than intradermal C. However, no significant difference was revealed between the two techniques for any time period. Our hypothesis—that the use of 4/0 polypropylene would be superior to 4/0 poliglecaprone 25 for intradermal suturing, because, as it is removed earlier, it would probably cause less skin irritation at the wound area and achieve better healing outcomes—has not been confirmed. Wound healing was better with polypropylene than with poliglecaprone 25 in the second po. month, but not afterwards, even if these differences were not significant. However, even if the use of polypropylene sutures in intradermal suturing with clips has not been proved to be better, it was found to have a comparable outcome to the use of poliglecaprone 25 with burying of the knots and its performance was easier and quicker. Its main disadvantage is the need to use dressings and Elizabethan collars to avoid early removal and wound dehiscence.

## 5. Conclusions

In conclusion, polypropylene was found to be a safe and effective suture material for use in intradermal suture patterns with clips in dogs and to have an easy and quick application. However, its earlier removal from wounds than poliglecaprone 25 was not associated with a beneficial effect on wound healing and scar appearance. Both suture materials are suitable for use in intradermal suture techniques in dogs.

## Figures and Tables

**Table 1:** Time (minutes) required for completion of each technique, the difference being significant (*p* < 0.001).

Technique	Percentile 25	Median	Percentile 75	Mean	Standard Deviation
Intradermal B (poliglecarpone 25)	15′32″	15′37″	16′55″	16′13″	1′30″
Intradermal C (polypropylene)	13′39″	13′48″	14′05″	13′33″	0′44″

**Table 2:** Median scores for the total cosmetic evaluations (interquartile ranges in brackets) for each time period (1–10 visual analogue scale; 1: excellent cosmetic result, 10: bad cosmetic result).

Technique	Period A 1st–8th po.d.	Period B 9th–21st po.d.	Period C 22nd–63rd po.d.	Period D 64th–180th po.d.	Period E 181st–365th po.d.
Intradermal B (poliglecaprone 25)	4.0 (4.0–4.5)	2.8 (2.0–3.0)	2.0 (1.8–3.0)	1.0 (1.0–2.0)	1.0 (1.0–2.0)
Intradermal C (polypropylene)	4.0 (3.3–4.5)	2.0 (1.0–3.0)	1.8 (1.0–2.0)	1.0 (1.0–2.0)	1.3 (1.0–2.0)

**Table 3:** Median values or scores for the clinical evaluations (interquartile ranges in brackets) for each time period.

Technique	Period A 1st–8th po.d.	Period B 9th–21st po.d.	Period C 22nd–63rd po.d.	Period D 64th–180th po.d.	Period E 181st–365th po.d.
Skin thickening (in mm, measured with a skin caliper)					
Intradermal B (poliglecaprone 25)	1.53 (1.03–1.85)	0.65 (0.35–0.75)	0.38 (0.05–0.75)	0.0	0.0
Intradermal C (polypropylene)	1.40 (1.0–1.70)	0.58 (0.30–1.0)	0.30 (0.10–0.50)	0.0 (0.0–0.40)	0 (0.0–0.30)
Erythema (in mm, measured with an electronic caliper)					
Intradermal B (poliglecaprone 25)	0.30 (0.43–0.60)	0.0	0.0	0.0	0.0
Intradermal C (polypropylene)	0.32 (0.0–0.60)	0.0	0.0	0.0	0.0
Scar width (in mm, measured with an electronic caliper)					
Intradermal B (poliglecaprone 25)	0.52 (0.43–0.60)	0.51 (0.44–0.60)	0.45 (0.37–0.60)	0.42 (0.29–0.56)	0.35 (0.23–0.48)
Intradermal C (polypropylene)	0.56 (0.47–0.67)	0.53 (0.45–0.60)	0.41 (0.27–0.57)	0.56 (0.41–0.66)	0.30 (0.24–0.48)
Hyperpigmentation (score 0–3)					
Intradermal B (poliglecaprone 25)	0.0	0.0	1.0 (0.0–1.0)	0.00 (0.0–1.0)	0.50 (0.0–1.0)
Intradermal C (polypropylene)	0.0	0.0	0.00 (0.0–1.0)	0.0 (0.0–1.00)	1.0 (0.0–1.0)
Total clinical evaluation (score 0–12)					
Intradermal B (poliglecaprone 25)	4 (4–5)	3 (2–4)	3 (2–4)	2 (1–3)	1 (0–4)
Intradermal C (polypropylene)	4 (4–5)	3 (2–4)	3 (2–4)	2 (1–3)	2 (1–3)

**Table 4:** Median u/s estimated wound area (in mm^2^) of the ultrasonographic evaluations (interquartile ranges in brackets) for each time period. The differences for periods A, B and C are statistically significant (*p* = 0.029, 0.025 and 0.011, respectively).

Technique	Period A 1st–8th po.d.	Period B 9th–21st po.d.	Period C 22nd–63rd po.d.	Period D 64th–180th po.d.	Period E 181st–365th po.d.
Intradermal B (poliglecaprone 25)	8.60 (7.13–10.31)	4.93 (4.39–5.74)	3.23 (2.67–4.26)	2.15 (1.45–2.90)	1.45 (0.70–2.21)
Intradermal C (polypropylene)	9.50 (7.87–11.20)	4.54 (3.55–5.50)	2.86 (2.20–3.65)	1.89 (1.42–2.57)	1.34 (0.80–2.24)

**Table 5:** Median values or scores for the histological evaluations (interquartile ranges in brackets) for each time period.

Technique	Period A 1st–8th po.d.	Period B 9th–21st po.d.	Period C 22nd–63rd po.d.	Period D 64th–180th po.d.	Period E 181st–365th po.d.
Inflammation (score 0–3)					
Intradermal B (poliglecaprone 25)	2 (2–3)	2 (2–2)	1 (0–2)	0 (0–1)	0 (0–0)
Intradermal C (polypropylene)	2 (2–3)	3 (1–3)	1 (0–1)	0 (0–0)	0 (0–0)
Epithelial thickness (number of times by which scar epithelial thickness was greater than that of the adjacent healthy epidermis)					
Intradermal B (poliglecaprone 25)	2.5 (2.0–3.0)	2.0 (2.0–3.0)	1.6 (1.5–2.0)	1.2 (1.0–1.3)	1.0 (1.0–1.1)
Intradermal C (polypropylene)	2.5 (2.0–2.5)	2.0 (2.0–3.0)	1.4 (1.2–1.5)	1.1 (1.0–1.3)	1.0 (1.0–1.2)
Scar width (in mm)					
Intradermal B (poliglecaprone 25)	0.54 (0.45–0.68)	0.56 (0.45–0.68)	0.59 (0.50–0.90)	0.45 (0.41–0.54)	0.45 (0.41–0.45)
Intradermal C (polypropylene)	0.68 (0.54–0.68)	0.63 (0.54–0.81)	0.52 (0.45–0.54)	0.45 (0.36–0.68)	0.45 (0.36–0.45)
Total histological evaluation (score 0–15)					
Intradermal B (poliglecaprone 25)	7 (6–8)	5 (5–6)	3 (2–5)	1 (0–2)	0 (0–0)
Intradermal C (polypropylene)	7 (6–8)	7 (5–7)	3 (2–4)	1 (0–2)	0 (0–3)

## Supplementary Materials

The following supporting information can be downloaded at: https://www.mdpi.com/article/10.3390/vetsci10020105/s1, Table S1: Scoring system for clinical examinations; Table S2: Scoring system for histological examinations.
